# Identification of a potential bioinformatics-based biomarker in keloids and its correlation with immune infiltration

**DOI:** 10.1186/s40001-023-01421-y

**Published:** 2023-11-02

**Authors:** Zihan Li, Chuwei Zhang, Qingrong Zhang, Yipeng Dong, Xinyu Sha, Ming Jiang, Jun Yan, Wenmiao Wang, Houqiang Li, Yi Zhang, You Lang Zhou

**Affiliations:** 1 Department of Burn and Plastic Surgery, Affiliated Hospital of Nantong University, Nantong, China; 2 Nantong University, Nantong, China; 3 The Hand Surgery Research Center, Department of Hand Surgery, Affiliated Hospital of Nantong University, Nantong, China; 4 Department of Thoracic Surgery, Affiliated Hospital of Nantong University, Nantong, China; 5 Institute of Burn Research, Southwest Hospital, State Key Lab of Trauma, Burn and Combined Injury, Chongqing Key Laboratory for Disease Proteomics, Third Military Medical University (Army Medical University), Chongqing, China

## Abstract

Keloid formation is a pathological consequence resulting from cutaneous irritation and injury, primarily attributed to excessive collagen matrix deposition and fibrous tissue proliferation. Chronic inflammation, left uncontrolled over an extended period, also stands as a substantial contributing factor. The precise mechanisms underlying keloid formation remain unclear. Therefore, this study aimed to identify key genes for diagnostic purposes. To achieve this, we used two Gene Expression Omnibus (GEO) data sets to identify differentially expressed genes. We identified one particular gene, homeobox C9 (*HOXC9)*, using a thorough strategy involving two algorithms (least absolute shrinkage and selection operator and support vector machine-recursive feature elimination) and weighted gene co-expression network analysis. We then assessed its expression in normal and keloid tissues. In addition, we explored its temporal expression patterns via Mfuzz time clustering analysis. In our comprehensive analysis, we observed that immune infiltration, as well as cell proliferation, are crucial to keloid formation. Thus, we investigated immune cell infiltration in the keloid and normal groups, as well as the correlation between *HOXC9* and these immune cells. It was found that *HOXC9* was closely associated with the immune microenvironment of keloids. This shows that *HOXC9* can serve as a potential biomarker and therapeutic target for keloids.

## Introduction

Keloid is an aberrant tissue characterized by overgrowth that arises after the healing of skin injuries or wounds, often for unknown reasons. Keloids can form over months or years and are linked to excruciating pain, inflammation, and different physical and psychosocial symptoms, such as pruritus [1]. The back, anterior earlobes, shoulders, and chest are the most prevalent sites for keloid formation, with the latter two being especially frequent due to higher strain. [2].

During the wound-healing process, myofibroblasts and fibroblasts are the primary mediators of collagen matrix remodeling and formation. These cells form an extracellular matrix with increased rigidity [3]. Different types of glycoproteins, collagens, and glycosaminoglycans form the keloid matrix [4]. Over time, the keloid matrix's overproduction of type III collagen is replaced by a significant proportion of type I collagen. Fibers in keloids are typically larger compared with those in normal skin [5]. Due to their morphology and clinically aggressive behavior, keloids caused by dermal trauma are frequently regarded as a form of non-specific aetiologic and benign fibroproliferative reticular skin tumor. The clinical features and morphology of these entities bear resemblance to neoplastic cutaneous tumors, exhibiting uncontrolled growth, excessive vascularization, and tissue invasion [6, 7].

The immune microenvironment is a pivotal factor in keloid formation. Keloids can form as a result of an exaggerated inflammatory response to tissue injury. This pathological state is characterized by an elevated number of inflammatory cells, particularly T cells, macrophages, and mast cells, within keloid tissues to varied extents [8]. These cells secrete a diverse array of cytokines/chemokines, including interleukins (ILs) and transforming growth factor β1, which are crucial inflammatory response-related mediators [9].

To date, the exact pathophysiology of keloid formation remains unclear; however, it is thought to be caused by skin tension [10], tissue hypoxia [11], chronic inflammation [12], autoimmunity [13], and genetic factors [14]. No effective treatment is currently available, and keloids often recur even after monotherapies, such as surgical removal [2]. As a result, identifying multiple biomarkers for this illness could potentially enhance patients' prognoses.

Cellular protein expression is critically involved in determining cellular development status [15]. Gene expression encompasses mRNA and protein transcription, translation, and turnover. The link between mRNA and protein levels affects translation and degradation processes, which are important for regulating transcription, mRNA stability, and gene expression [16]. Gene expression is crucial for the development and sustainability of several biological activities, including cell proliferation, cell cycle, and apoptosis, and irregularities in it can lead to disorders, such as cancer [17]. Therefore, understanding these molecules’ biological activities in keloids is crucial for identifying potential treatment applications. This study aimed to uncover potential markers to distinguish patients with keloids from those who did not have keloids. The intramodular hub gene and module eigengene (ME) can be used to summarize gene clusters and find highly linked genes using the weighted gene co-expression network analysis (WGCNA) technique. In addition, it calculates module membership (MM) measures and establishes connections between modules and external sample attributes using the eigengene network methodology. Gene screening techniques that use this network-based methodology can identify prospective therapeutic targets and aid in the development of effective therapies [18]. In this study, we used WGCNA as one of the methods to screen key genes for disease.

In this study, we integrated two data sets sourced from the GEO database to augment the sample size. We compared gene expression profiles between the normal and disease groups and identified the differentially expressed genes (DEGs). WGCNA was then used to obtain hub genes, which were then intersected with the DEGs. The LASSO technique was used to further screen the genes selected to identify a key gene. We analyzed the expression of this key gene in normal and keloid tissues, examined its temporal changes concerning other genes using Mfuzz time clustering [19], and recognized the most relevant gene cluster. Finally, we examined immune cell infiltration in the keloid and normal groups, as well as the correlation between this key gene and immune cells.

## Materials and methods

### Data sources and quality control

We retrieved two data sets of original expression profile data from the NCBI’s GEO database, GSE7890 [20] and GSE83286 [21], for this investigation. These data sets were associated with the GPL570and GPL19612 platforms, respectively. Smith J. et al. (https://www.ncbi.nlm.nih.gov/geo/query/acc.cgi?acc=GSE7890) collected samples from both normal dermis and keloids of adult males and females in the GSE7890 data set. From these samples, primary cells were collected for further culture and analysis. To ensure data integrity, samples treated with hydrocortisone were excluded from our study, leaving us with a total of ten samples. The GSE83286 data set contained three samples of keloid lesions from human earlobe keloids and three samples of normal skin from earlobe piercing. Guo L. et al. provided (https://www.ncbi.nlm.nih.gov/geo/query/acc.cgi?acc=GSE83286). In addition, we used the GSE145725 [22] (https://www.ncbi.nlm.nih.gov/geo/query/acc.cgi?acc=GSE145725) data set based on GPL16043, which contained ten normal fibroblasts and nine keloid fibroblasts, for validation.

### Preparation and identification of DEGs

To annotate two series matrix files, several official gene symbols from the data table of the microarray platform were employed. The ensuing gene expression matrix files were then combined into a single file. The expression data from the three data sets were then batch-normalized using the “sva” R package. The “limma” R package was used to perform DEG analysis on the generated gene expression matrix files. To identify DEGs, we used a cutoff threshold of *P* < 0.05 and |logFC|> 1.5. Based on the findings of the DEG analysis, volcano plots and heat maps were generated.

### Enrichment analysis

#### Gene ontology (GO)

The “clusterProfiler” package was used in R to perform GO enrichment analysis. To find enriched GO terms, we set a threshold of *P* value < 0.05 and an adjusted *P* value < 0.05.

### protein–protein interaction (PPI) network analysis

Utilizing the Search Tool for the Retrieval of Interacting Genes (STRING) database (http://www.string-db.org), the DEGs identified in this investigation were used to develop a PPI network. The minimal interaction score needed was set at 0.4.

### WGCNA network construction and module identification

Using the “WGCNA” R package, we analyzed co-expression networks [23]. Initially, we examined the samples for potential outliers or missing values and processed or removed them as necessary. We then used an automatic network construction function to develop the co-expression network. The soft thresholding power was determined using R's "pickSoftThreshold" function. After that, we performed a hierarchical clustering analysis (minModuleSize = 60) after converting the adjacency matrix into a topological overlap matrix (TOM). Similar modules were merged based on commonalities discovered using the dynamic tree-cut function. We computed the ME, which reflects the module's first principal component and characterizes the module's expression pattern. In addition, we calculated the MM to determine the relationship between genes and MEs. This metric serves as an indicator of the dependability of genes in modules and supports the identification of modules related to clinical features. Following that, gene significance (GS) was calculated to link modules to clinical features, and the resulting data were shown. Finally, we retrieved pertinent gene information from the corresponding modules for further study.

### Screening biomarkers

To identify key genes, we selected appropriate module genes and intersected them with DEGs. We then performed minimum absolute value convergence using the glmnet package and variable selection using LASSO. Using the cv. glmnet function in the glmnet package, a model cross-validation analysis was performed with the following parameters: Family = “binomial,” alpha = 1, and nfolds = 10. The “svmRadial” method in R was also used to implement another algorithm, SVM–RFE, and a model cross-validation analysis was carried out, for further analysis and identification of the key gene.

### Verification of biomarkers

The receiver operating characteristic (ROC) approach was used with the R package "pROC" to evaluate the sensitivity and specificity of the genes identified through SVM–RFE and LASSO analyses. We collected skin and keloid tissues from patients having scar excision surgery to isolate fibroblasts and primary keratinocytes for the expression profile data.

### Cell- and tissue-specific gene expression analysis

The Human Protein Atlas (HPA) database (https://www.proteinatlas.org/) is a web-based resource that contains data from transcriptomics, proteomics, and system biology. It facilitates the identification of target gene expression in various cells, tissues, and organs and provides detailed maps of these biological components. The database includes information on patient survival curves as well as the protein expression of normal tissues as well as diseased tissues. Another useful online tool for retrieving and comparing gene expression patterns across several animal species is the Bgee database (https://bgee.org/). The gene expression levels of normal tissues can be compared using the expression data provided on this website, which are derived exclusively from healthy tissues utilized in earlier studies.

### Mfuzz time clustering analysis

Genes within our samples were clustered using Mfuzz, an R tool created for the soft clustering of microarray data, based on their expression profiles to establish unique gene sets. After removing outliers or missing values from this analysis, we determined that there were 30 gene clusters. Subsequently, we used the “GSVA” method to calculate the enriched scores for these gene clusters. This enabled us to assess the variations in scores across several gene clusters between the control and scar groups. To investigate the expression features of the three genes indicated above further, we calculated and plotted shifts in marker gene expression across multiple gene clusters and performed correlation tests.

### PPI network

To investigate the gene clusters selected earlier, we conducted protein interaction network analyses. Using the STRING database, we set the confidence levels for clusters 1 and 5 at ≥ 0.07 and ≥ 0.09, respectively. We used Cytoscape (3.7.2) to visualize the results.

### Analysis of immune cell infiltration and differences

Each tissue’s immune infiltration was examined using CIBERSORT, a program that analyzes gene expression profiles to determine the cell composition of complex tissues. In addition, we assessed the relative number of immune cells in our samples using the ssGSEA algorithm. We previously acquired comprehensive gene sets related to immune cells for this study. The enrichment scores we calculated for the samples served as an estimate of the immune cell infiltration level. Using these results, we constructed heat maps of immune cell distributions. Subsequently, we conducted comparisons to identify variations in the expression patterns of distinct immune cells between the keloid and normal groups.

### Analysis of correlation between biomarkers and immune cells

Using the R packages “tidyverse,” “reshape2,” and “limma,” we conducted a correlation analysis between immune cells and marker genes. We formatted the data using the “ggplot2” package to effectively present the results.

## Results

### Identification of DEGs

**Fig. 1:**
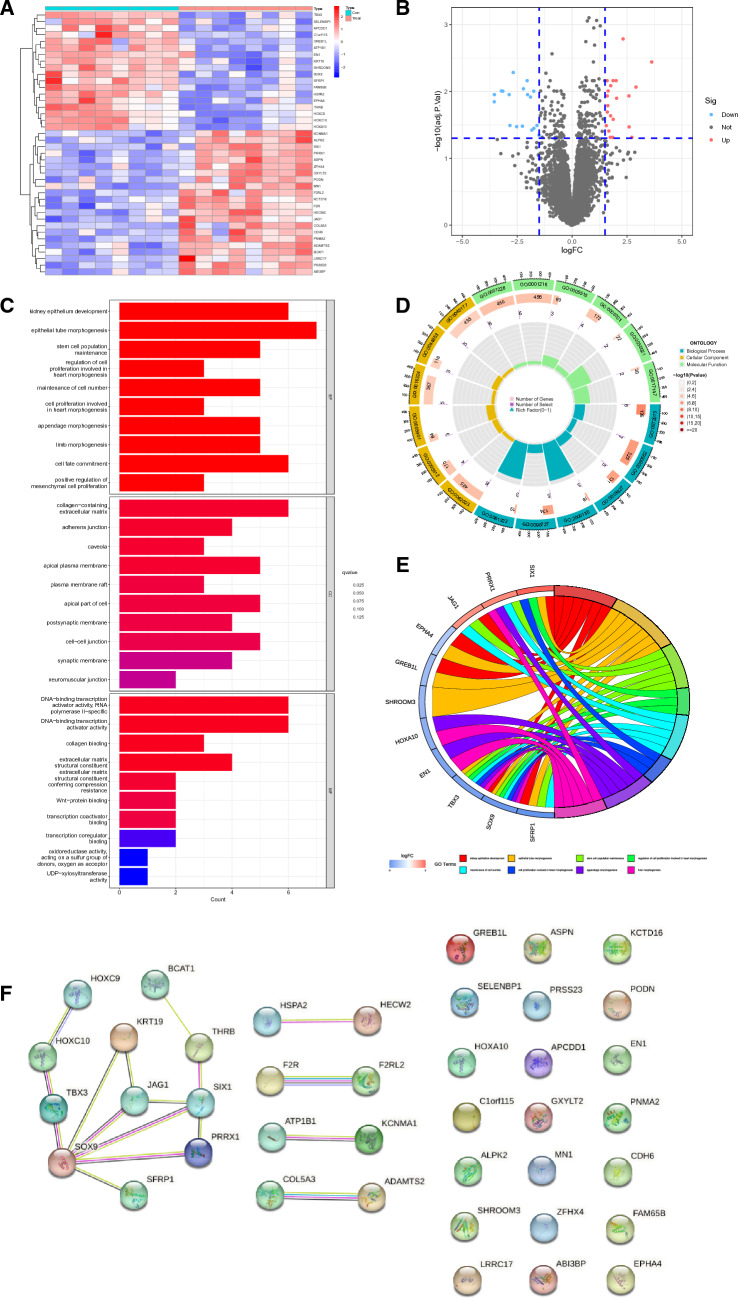
Identification of differentially expressed genes (DEGs) and their enrichment. **A** Heatmap visualizes the DEGs, with red indicating high expression and blue indicating low expression. Deeper colors represent greater differences. **B** Volcano plot shows DEG distribution. Blue and red dots show low- and high-expressed DEGs, respectively. **C** Gene ontology (GO) enrichment analysis of DEGs. Every GO term’s number of genes is shown on the horizontal axis. **D**, **E** Circlize and circos diagrams of the GO enrichment analysis of DEGs. **F** Protein–protein interaction network of DEGs

We obtained the GSE7890 and GSE83286 data sets from the GEO database, which have been previously studied. After correcting for batch effects, we combined them. We identified 40 DEGs in total using the "limma" R package, with 22 upregulated and 18 downregulated genes. The heatmap shows all of the DEGs (Fig. 1A). In addition, Fig. 1B shows DEG distribution, with red and blue dots showing the same expression characteristics as previously.

### DEG PPI and function prediction

We conducted GO analyses comprising biological processes (BPs), cellular components (CCs), and molecular functions (MFs) to acquire insights into the probable functionalities of these 40 DEGs. These DEGs were shown to be enriched in BPs such as cell proliferation implicated in heart morphogenesis, positive control of mesenchymal cell proliferation, and stem cell population maintenance, according to the findings. In addition, they were linked to CCs, such as adherens junctions, the apical plasma membrane, and the extracellular matrix, which contains collagen. Using MF analysis, it was discovered that DEGs had a strong correlation with collagen binding, RNA polymerase II-specific activity, DNA-binding transcription activator activity, and extracellular matrix structural constituents (Fig. 1C, D). GOcircus (Fig. 1E) additionally demonstrated that seven genes were enriched in GO terms.

We developed a PPI network using the STRING database (http://www.string-db.org), an online database, based on the interactions between these genes. These interactions are presented in Fig. 1F. For upcoming research, genes that do not interact with others were eliminated.

### WGCNA and key module identification

**Fig. 2:**
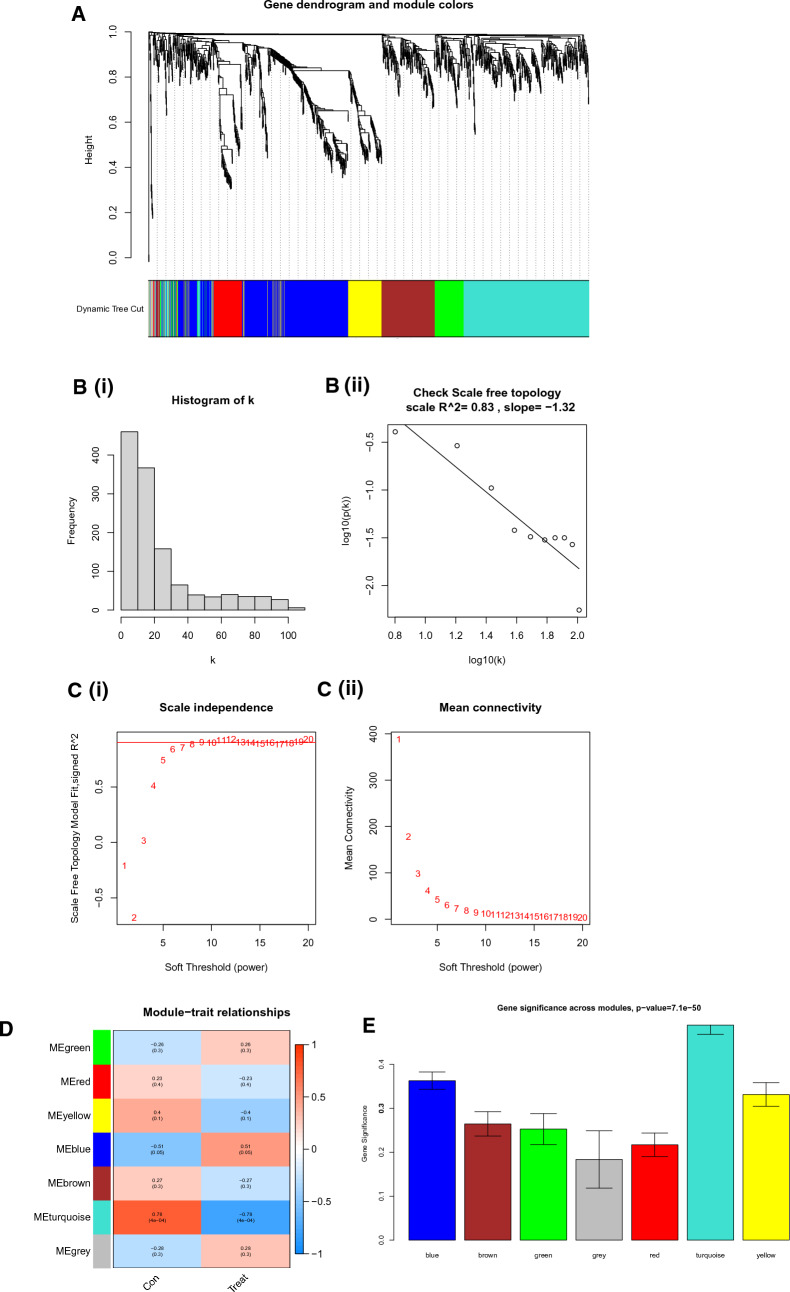

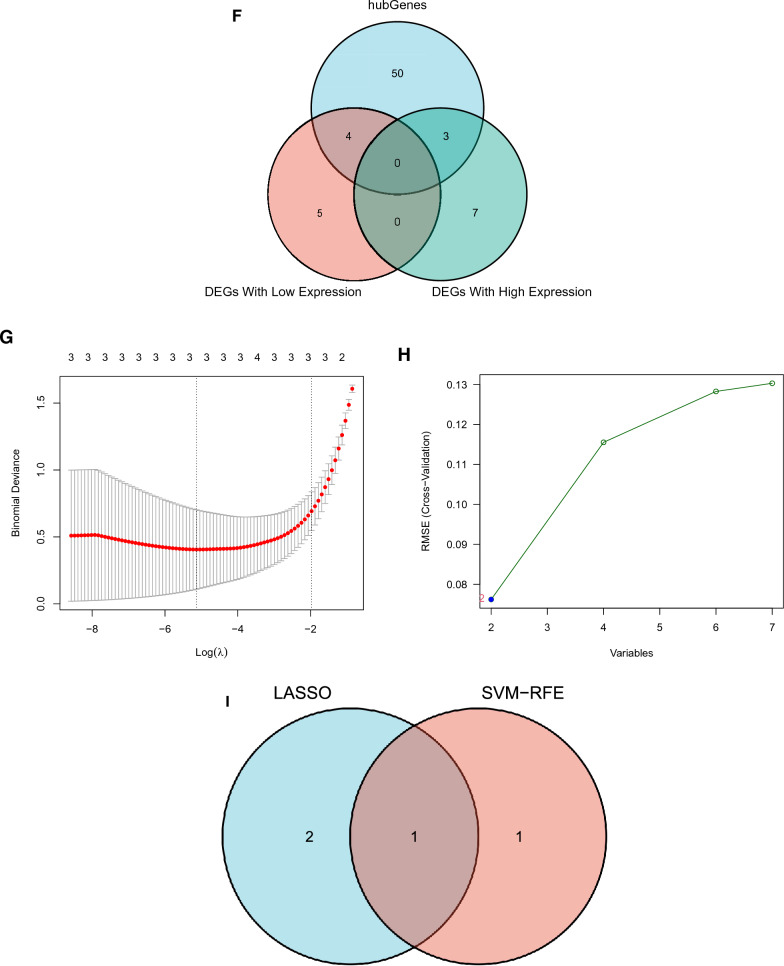
Screening biomarkers. **A** Weighted gene co-expression network analysis (WGCNA) of genes. Branches are distinguished by different colors, representing seven distinct modules. **B,** **C** Evaluation of the scale-free topology criterion with R2 = 0.83 and the soft thresholding power = 7. **D** Correlation plot of WGCNA modules with trait attributes. Rows and columns represent modules and trait attributes, respectively. Red denotes a positive correlation, whereas blue denotes a negative correlation. **E** Significance of each module’s genes. **F** Venn diagram showing the intersection genes of DEGs and the turquoise module. **G** Cross-validation for choosing LASSO regression model tuning parameters. Three genes were examined, and dotted vertical lines were created at the optimal levels. **H** Screening genes using the SVM–RFE algorithm. The number of genes with the optimal accuracy is represented by the blue dots. **I** Venn diagram showing the discovery of significant genes using LASSO and SVM analyses

Using WGCNA, we identified co-expression modules of genes with high degrees of co-expression and topological overlap resemblance Initially, the samples were clustered, and a sample clustering tree was generated (Fig. 2A). A scale-free network was successfully designed using the R function “pickSoftThreshold” by establishing a soft thresholding power of 7 according to the scale-free topology criterion, resulting in an R2 value of 0.83. The adjacency matrix was then converted into a TOM to display node similarity while accounting for weighted correlations (Fig. 2B, C). We merged similar modules after locating them using hierarchical clustering and dynamic tree-cut analysis. More ME, MM, and GS calculations were performed to link modules to clinical characteristics. The generated data were presented using heatmaps (Fig. 2D) and bar charts to underline the importance of each module. These results (Fig. 2E) revealed that the turquoise module gene, which was considered a key module, had the lowest *P* value.

### Screening biomarkers

We chose the turquoise module genes and intersected them with the DEGs based on earlier calculations before further screening the resulting genes (Fig. 2F). We identified three high-expressed and four low-expressed DEGs in the turquoise module. LASSO and SVM–RFE were then used. Cross-validation analysis in the LASSO regression model selected the optimal *λ* value, minimizing error and finding three keloid-related genes: *HSPA2*, *HECW2*, and *homeobox C9 (HOXC9*) (Fig. 2G). Using the SVM–RFE technique, two genes, *HOXC9* and *F2R*, were discovered similarly (Fig. 2H). Finally, using the intersection of the genes found by the two algorithms, we discovered HOXC9 to be the biomarker (Fig. 2I).

### Biomarker verification

**Fig. 3:**
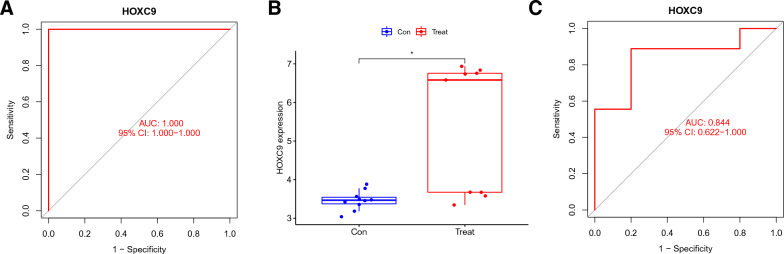
Biomarker verification. **A** *HOXC9*-related ROC analysis. **B**, **C** GSE145725 data set-based *HOXC9* validation

We used ROC analysis to determine *HOXC9*’s diagnostic value in the merged GSE7890 and GSE83286 samples. The high diagnostic value of *HOXC9* was indicated by its area under the curve value of 1.000 (Fig. 3A). To validate the reliability of these results, we selected the GSE145725 data set for verification, yielding a result of 0.844 for the gene (Fig. 3C). This finding further confirms the validity of our previous analysis.

### Expression of the biomarker in normal tissues

**Fig. 4:**
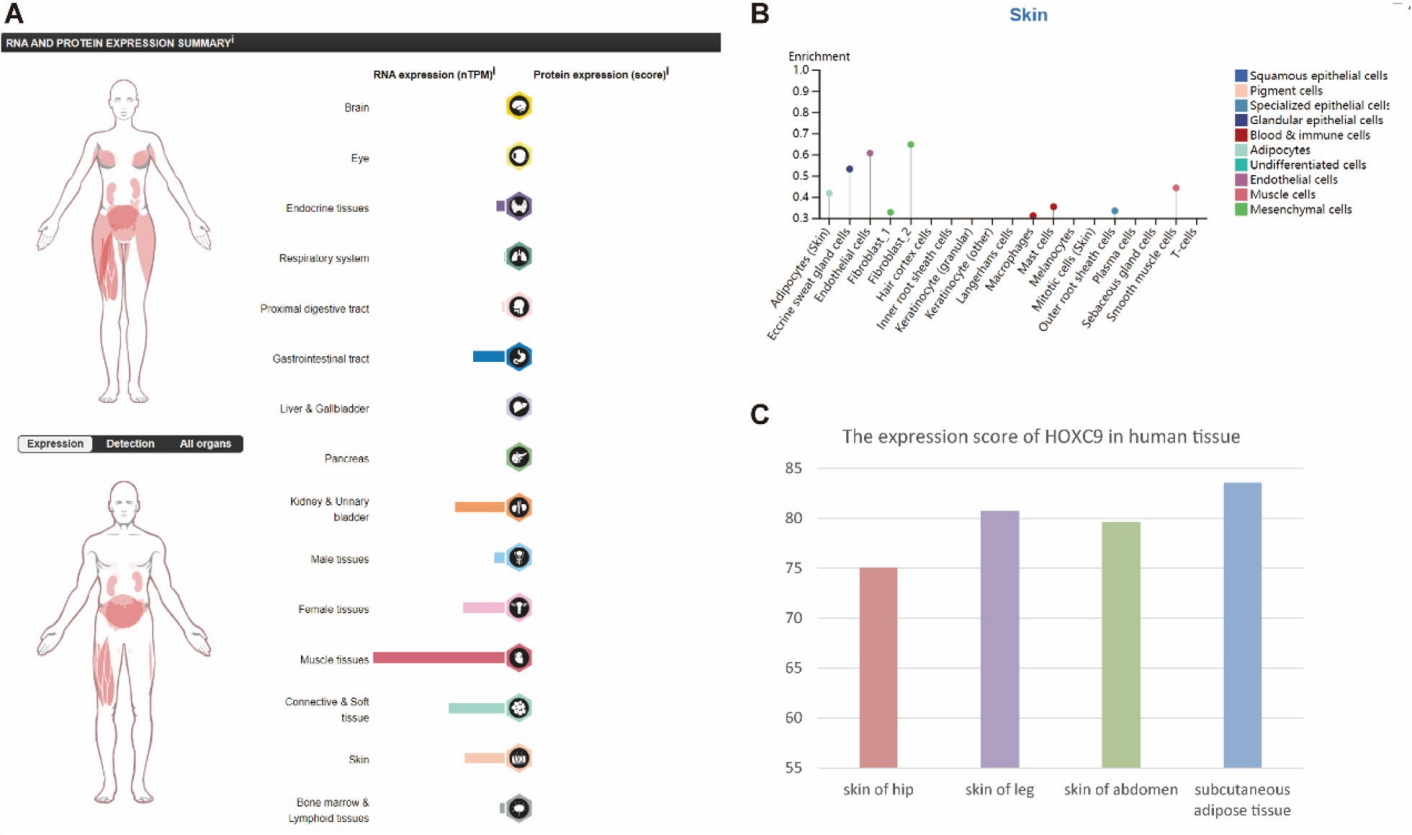
The biomarker's expression in normal tissues. **A** Human protein Atlas *HOXC9* expression. **B** HOXC9 expression in different skin cell types. **C** Human tissue *HOXC9* expression

To gain a comprehensive understanding of this biomarker, we examined its expression across the entire body (Fig. 4A) using data from the HPA database. We also investigated its enrichment profile in diverse skin tissue cell types. According to our findings, the following cell types expressed *HOXC9*: endothelial cells, smooth muscle cells, eccrine sweat gland cells, adipocytes, and fibroblast_2 (mesenchymal cells) (Fig. 4B). In addition, using the Bgee database, we evaluated its expression in multiple skin locations and the adjacent tissues of healthy persons, and a histogram of the results is shown in Fig. 4C. These results revealed that the differences in gene expression between the subcutaneous adipose and skin tissue were not particularly pronounced, suggesting its possible role in keloid formation.

### Temporal biomarker expression clustering and correlation analysis

**Fig. 5:**
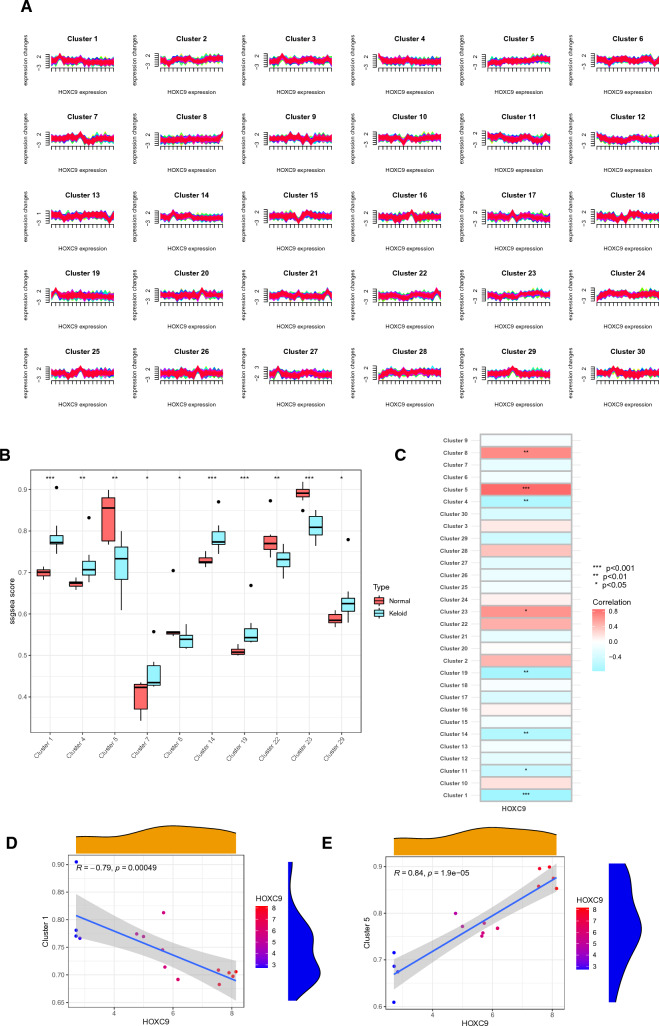

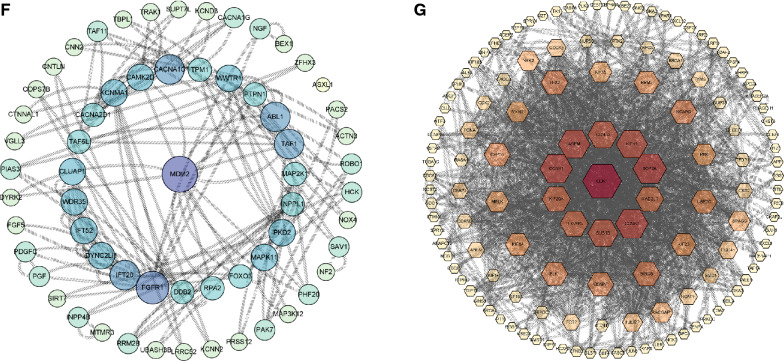
Time clustering of expression and correlation tests of biomarkers. **A** Changes in gene expression in 30 different gene clusters as marker gene expression increases. **B** Differences in enrichment scores between the keloid and normal groups. **C** Correlation of HOXC9 with different gene clusters. **D**, **E** Correlation of HOXC9 with clusters 1 and 5. **F**, **G** Protein–protein interaction network of gene clusters 1 and 5

Based on the expression pattern, we organized the genes into 30 distinct clusters. Subsequently, we explored the changes in *HOXC9* expression across these clusters, elucidating how the expression of other genes correlated with the biomarker levels (Fig. 5A). To delve deeper into the expression characteristics of the biomarker, we calculated the enrichment scores for each gene set in which the sample genes were located. We compared the scores and visualized the outcomes between the keloid and normal groups (Fig. 5B). The gene clusters with *P* < 0.05 are highlighted in this figure. Following this, correlation tests were conducted, which indicated that *HOXC9* displayed the highest level of positive association with gene cluster 5 and the most substantial negative correlation with cluster 1 (Fig. 5C). The relationships were subsequently shown (Fig. 5D, E), and two gene clusters were chosen for further research.

### Gene cluster PPI and core gene role

Using online tools, protein–protein interaction network analysis was performed on the two gene clusters stated above. Using Cytoscape software, we further constructed PPI networks for clusters 1 and 5. Cluster 1 had a total of 409 nodes and 439 identified edges, according to STRING, whereas Cluster 5 had 440 nodes and 770 identified edges. The final results are presented in Fig. 5F, G. Based on previous studies, *MDM2* and *CDK1*, the cluster's core genes, are linked to *HOXC9*. These key genes affect cell growth and immune infiltration. Due to the importance of immune infiltration in keloid formation, the immune microenvironment must be studied.

### Correlation between biomarker and immune cell infiltration

**Fig. 6:**
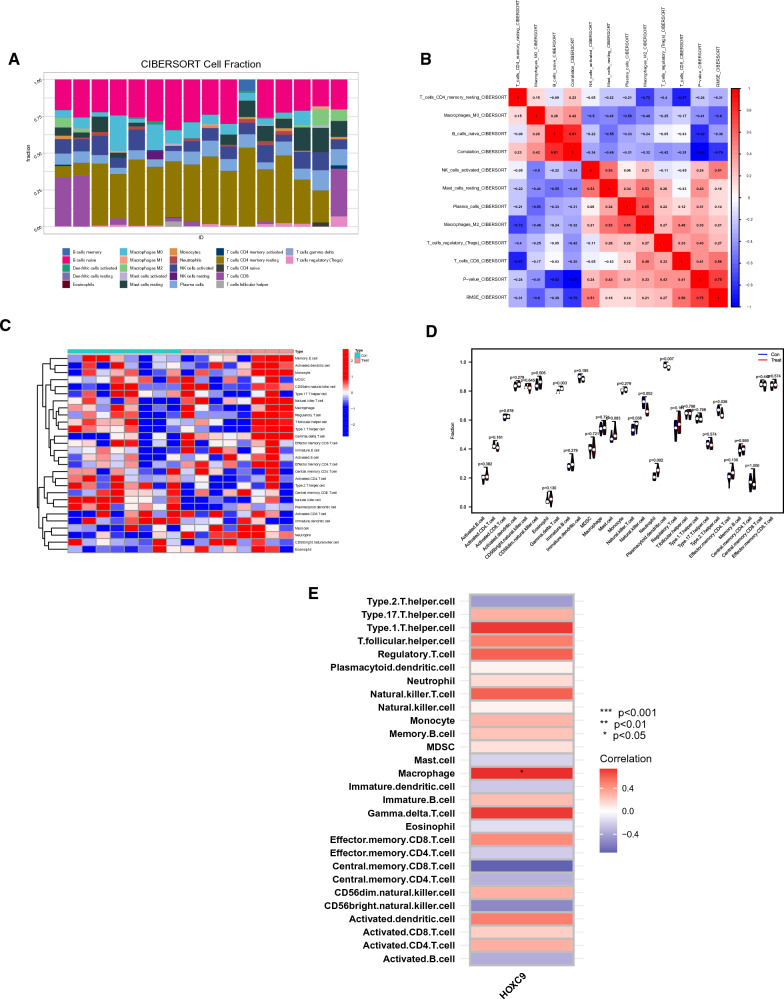
Immune cell infiltration and correlation between biomarkers and immune cells. **A** Infiltration of different immune cells in tissues. **B** Heatmap of immune cell infiltration. **C** Correlation heatmap of immune cells. Red indicates a positive correlation, blue indicates a negative correlation and darker colors denote stronger correlations. **D** Differences in different types of immune cells between the two groups of samples. **E** The correlation of HOXC9 with different immune cells

The surrounding microenvironment, in particular the immune microenvironment, plays a crucial role in the formation of keloid lesions. Uncertainty persists regarding the precise relationship between immune cells and keloid formation. We investigated the immune infiltration in several tissues using CIBERSORT, and the outcomes are shown in Fig. 6A. We generated a correlation heatmap of immune cells (Fig. 6B). In addition, we utilized the ssGSEA algorithm to investigate immune cell infiltration in the tissues. We made a heatmap of immune cells (Fig. 6C) to better visualize our results. Red denotes a positive correlation, blue denotes a negative correlation, and the intensity of the color denotes the strength of the association. The expression of certain immune cells was then investigated in keloid and normal tissues. Significant variations were found in the expression of type 2 T helper cells, gamma delta T cells, plasmacytoid dendritic cells, natural killer T cells, neutrophils, natural killer cells, and (Th2) cells (Fig. 6D). We conducted a correlation analysis to determine the association between immune cells and HOXC9, which demonstrated a positive relationship between HOXC9 and macrophages (Fig. 6E).

## Discussion

Keloids are characterized by the formation of scar tissue that extends beyond the original skin injury site, and they commonly occur in persons who are predisposed to this condition [24]. The etiology of these benign fibroproliferative disorders is uncertain. They are further distinguished by blood vessel hyperproliferation [25, 26], epidermal thickening, increased mesenchymal cell density [27], thick and dense hyalinized collagen fibers [28], and excessive fibronectin proliferation [29]. Keloid tissue has an extensive extracellular matrix meshwork [27] due to excessive collagen deposition and growth [30] and irregular fiber distribution. In contrast, normal skin possesses collagen bundles parallel to the epidermis [31].

Currently, a variety of methods, including compression therapy, corticosteroid injections, and surgical procedures, are used in clinical practice for the treatment of keloid scars. However, the recurrence rates of keloids remain high [12]. Keloids can potentially impair limb function and affect patients' quality of life in some cases. Our study aimed to discover prospective targets for keloid therapy. We found that DEGs were abundant in BPs, CCs, and MFs associated with keloid fibroblasts by means of differential expression analysis. In terms of MF, many DEGs were involved in DNA replication activities and the construction of the extracellular matrix. In BPs, we observed enhanced cell proliferation abilities. In addition, their positive regulation of mesenchymal cells and the maintenance of stem cells were emphasized. These findings suggest that these DEGs may lead to the highly activated state of cells in keloid tissue, resulting in excessive proliferation and increased deposition of the extracellular matrix. Furthermore, some skin cells, such as dermal fibroblasts, can secrete extracellular matrix components, such as collagen. As the proliferation ability and quantity of cells increase, so does the extracellular matrix increase, including collagen. Moreover, the cells become tightly connected, contributing to the tough texture of keloids. Cellular component analysis further supports these findings.

We used the LASSO algorithm and WGCNA to explore potential biomarkers. Using this comprehensive approach, we discovered a key gene, *HOXC9*, that was related to keloids in the GSE7890 and GSE83286 data sets. In normal tissues, our data demonstrated that the *HOXC9* expression pattern was similar in the skin and its adjacent tissues. However, *HOXC9*'s specific expression pattern in keloid tissues indicated its crucial role in keloid formation regulation.

The homeobox gene family contains the protein-coding gene *HOXC9*. Homeobox genes encode a highly conserved group of transcription factors known for their involvement in morphogenesis during the development of multicellular organisms. *HOXC9* has been linked to different disorders, despite not previously being connected to keloids. It is well-known that *HOXC9* plays a crucial function in regulating vascular morphogenesis and preserving endothelial cell quiescence[32]. *HOXC9* may have an impact on angiogenesis, which is crucial for the formation of keloids. In addition, *HOXC9* can modulate these processes via inhibiting interleukin (IL)-8 [33]. Studies have linked low expression of *HOXC9* to various conditions, including lymph node metastasis, papillary thyroid cancer, and Hashimoto’s thyroiditis [34]. Conversely, high *HOXC9* expression has been observed in human gastric cancer [35] and non-small cell lung cancer (NSCLC) [33]. It might be linked to a poor tumor prognosis depending on the situation. Overexpression of this gene promotes the invasion and development of NSCLC cells. It has also been determined that the circCENPF/has-miR184 axis is a potential target for the development of cancer cells [36]. *HOXC9* overexpression can also promote the development of stem cell- and metastasis-like cells in gastric cancer cells [37]. The inhibition of the DAPK1/RIG1/STAT1 axis can lead to interferon-gamma resistance [38]. *HOXC9* overexpression enhances invasiveness but decreases proliferation in various breast cancer cell lines. This is suggestive of a switch from the proliferative to the invasive phase of cancer cell development [39]. Consequently, *HOXC9* may play a crucial role in cell proliferation, cell migration, angiogenesis, and other activities significantly linked to keloid formation.

Using a soft cluster analysis, we identified two gene sets closely associated with changes in *HOXC9* expression. The key genes in these two sets were *CDK1* and *MDM2*. According to reports, *MDM2* can activate fibroblasts, resulting in fibrosis [40]. It may also contribute to the emergence of inflammation [41]. *CDK1* is necessary for controlling cell division during the G2/M phase and mitosis. Its overexpression has been linked to the development and progression of various cancers [42]. When *THRIL* is overexpressed, *CDK1*, which is involved in cellular motility and proliferation, is downregulated [43]. Several studies and analyses have connected *CDK1* to immune infiltration in lung adenocarcinoma [44], colorectal cancer [45], and hepatocellular carcinoma [46, 47]. These findings illustrate that these two key genes, which have a high correlation with *HOXC9*, are involved in cell proliferation and are intimately related to immune infiltration. Keloids exhibit a close relationship with the immune microenvironment, and genes related to immune infiltration and immune cell abnormalities may underlie keloid formation. Therefore, it is imperative to investigate the immune microenvironment within keloid tissues.

As inflammatory proliferative fibrous diseases, immune infiltration is critically involved in keloid formation. Previous studies have underscored the substantial role of various inflammatory cells in the wound-healing process. For instance, the equilibrium between the M1 and M2 phenotypes is critical for controlling inflammation and tissue healing, which are regulated by macrophages. High macrophage infiltration, mostly of the M2 type, has been found in the keloid specimens [48]. Similarly, T cells are vital contributors. T-cell subset diversity contributes to these cells' complex role in the keloid formation process. For instance, it is known that Th2 cells release IL-4 and IL-13, which can both boost collagen synthesis and metabolism and cause the accumulation of reticular fibrin [49]. Th1 cells can attenuate tissue fibrosis by secreting IFN-γ, which suppresses fibroblast proliferation and decreases the expression of type I and III collagen genes [50]. Treg cells, known for regulating other effector T cells, may directly influence collagen deposition in keloids. Furthermore, the substantial increase in mast cell population in keloids implies their integral role in keloid formation [51], with their numbers and activation status being positively y associated with the degree of keloid formation [52]. By secreting IL-4, VEGF, and basic fibroblast growth factors via the PI-3 K/Akt signaling pathway, mast cells can enhance fibroblast proliferation [52, 53]. Furthermore, it is worth noting that inflammatory chemicals play an important role as key mediators in the execution of immune cell activities. TGF-β levels are increased by Th2 cytokines, such as IL-4 and IL-13, leading to fibrosis. Hence, blocking Th2 cytokines represents a potential treatment avenue for keloids [54]. According to a previous study, keloid tissue's growing margin (perilesional region) also contains IL-17. It can encourage the production of SDF-1 in fibroblasts, which in turn facilitates the recruitment of Th17 cells that produce IL-17, establishing a positive feedback loop [55]. In addition, IL-17 greatly increases STAT3 and HIF-1 expression in healthy fibroblasts, impairing autophagy, which is linked to an increase in necroptosis and fibrosis [56]. This finding shows that suppressing STAT3 might be a feasible keloid therapy strategy [55].

We investigated the degree of immune cell infiltration in the samples from the GEO database, since immune infiltration contributes significantly to the formation of scarring. According to our findings, there were significant variations in the levels of neutrophils, natural killer T cells, gamma delta T cells, plasmacytoid dendritic cells, Th2 cells, and natural killer T cells between the two groups. A previous study has shown that an abnormal T-cell response triggers keloid progression [57]. In our research, we delved into the link between *HOXC9* and immune cells, and the findings are presented in the preceding section. *HOXC9* has been shown to exhibit a certain relationship with T-cell activity [58], which is a factor that has previously been discussed in the context of keloids. Collectively, our findings underscore the critical function of *HOXC9* in keloids. However, its functional mechanism and association with immune infiltration remains unclear, warranting further investigation.

In our study, we not only compared the differences in *HOXC9* expression between normal and keloid tissues but also highlighted the aberrant *HOXC9* expression in keloids by evaluating its expression in normal tissues. Given the findings of differential and enrichment analyses, we hypothesize that *HOXC9* may promote keloid formation by promoting cell proliferation and migration, increasing extracellular matrix deposition, and enhancing angiogenesis in keloid tissues. Furthermore, we identified key gene sets associated with the upregulation of *HOXC9*, further highlighting its connection to keloid formation and the immune microenvironment. Nevertheless, this study has some limitations. First, some differences were present between the samples. In this investigation, we chose GSE7890 and GSE83286 data sets and evaluated their combined results with GSE145725. These data sets, on the other hand, contain samples obtained from tissue samples or primary cells obtained from tissues. Consequently, variations between them could lead to differences in gene expression. Second, because the sample size is still too small, more microarray data must be evaluated to increase the results' reliability. Finally, further experimental validation, including cellular and animal experiments, is required to substantiate these findings. Our study findings suggest that *HOXC9* may influence the immune microenvironment of keloids, thereby promoting their growth. Subsequent experiments may include the establishment of animal models of keloid. These models can be used to observe tissue sizes and histological changes after reducing *HOXC9* expression to verify the effect of *HOXC9* on keloid growth. Furthermore, in cellular experiments, primary cells, such as fibroblasts and keratinocytes, can be cultured to investigate the effects of *HOXC9* reduction on cellular activities, including cell proliferation, migration, and apoptosis. In addition, keloid formation is influenced by environmental factors, such as inflammatory cell infiltration. The association between *HOXC9* and immune infiltration remains unclear [36]. Therefore, investigating whether reducing *HOXC9* expression can lead to improved immune cell infiltration. Gene therapy methods are gaining prominence, but their application in keloid treatment remains relatively rare. Validating the role and expression of *HOXC9* in keloids through various methods can pave the way for its clinical use. This approach offers a feasible method for keloid treatment.

## Conclusion

In this study, we performed bioinformatics analyses to detect key genes, such as *HOXC9*, which exhibited differential expression between normal and keloid tissues. Our findings demonstrate that *HOXC9* is critical in keloid formation and intricately linked to the keloid immune microenvironment. Therefore, it may serve as a valuable biomarker for keloid diagnosis and treatment. Our study findings not only provide valuable insights for future experimental investigations but also offer a novel avenue for clinical intervention.

## Data Availability

The genetic/RNA sequencing data for this study were obtained from the NCBI-Gene Expression Omnibus (GEO) database (https://www.ncbi.nlm.nih.gov/geo/).
